# Modeling the Geometry–Acoustics Dependence in Photoacoustic Resonators: A Toroidal Case Study

**DOI:** 10.3390/s26051496

**Published:** 2026-02-27

**Authors:** Enza Panzardi, Anna Lo Grasso, Valerio Vignoli, Ada Fort

**Affiliations:** 1 Department of Information Engineering and Mathematical Sciences, University of Siena, 53100 Siena, Italy; 2 Polytechnic Department of Engineering and Architecture, University of Udine, 33100 Udine, Italy

**Keywords:** photoacoustic gas sensors, acoustic resonator, toroidal geometry, FEM simulation, photoacoustic spectroscopy, resonance frequency, quality factor, resonator modeling

## Abstract

In this work we investigate the behavior of a toroidal photoacoustic resonator to provide compact, physics-guided analytical relationships that link its geometry to two key parameters: resonance frequency and quality factor. Finite-element data are combined with reduced-order analytical models to refine a corrected toroidal-resonance frequency model that accounts for effective propagation length and thermo-viscous effects. For the quality factor, a simple law motivated by a boundary-layer dissipation model is proposed. Derived models are validated by experimental tests performed using three 3D printed toroidal resonators in different sizes. Experimental results confirm the prediction both for the first and third resonance frequencies with an average relative error below 1%, outperforming cylindrical and uncorrected baseline models available in the literature. The results also confirm the predicted trend of the quality factor with respect to the torus’s minor radius, highlighting a direct relationship between the cross-sectional area and acoustic losses, which governs the balance between stored acoustic energy and thermo-viscous dissipation. Overall, the framework provides quick, interpretable design rules that reduce dependence on extensive finite-element method simulation campaigns for first-pass estimation of resonant behavior during the early design phase and guiding the optimization of high-performance PAS devices while preserving accuracy.

## 1. Introduction

Photoacoustic spectroscopy (PAS) is a versatile and highly sensitive technique widely used for trace gas detection in environmental, industrial, and medical applications [1,2]. Its compact implementation and capability to detect weakly absorbing species make it particularly attractive for portable gas sensing systems [3,4,5].

PAS is a subclass of optical spectroscopy that measures optical absorption indirectly through the generation of acoustic waves. The fundamental mechanism relies on the absorption of periodically modulated light by a sample, followed by non-radiative relaxation processes that lead to periodic local heating [6]. The released heat also follows a periodic pattern, inducing pressure oscillations in the surrounding medium which are detected as acoustic waves using sensitive microphones or piezoelectric transducers. The intensity of the generated acoustic wave is directly proportional to the concentration of the absorbing species, making PAS a powerful tool for trace gas analysis [7]. Its ability to detect weakly absorbing or optically opaque samples, support multi-component analysis, and operate in compact configurations makes it highly attractive compared to conventional optical methods.

With reference to gas detection applications, a conventional PAS system comprises a modulated light source, a photoacoustic cell containing the sample, an acoustic detection device, and signal processing electronics. The photoacoustic signal is typically weak in intensity since trace gas amounts absorb a minute fraction of energy. As a result, the detected signal requires accurate amplification and conditioning to ensure reliable detection [8,9]. In this context, the use of resonant photoacoustic cells is commonly employed for this purpose. Properly matching the modulation frequency with the acoustic eigenmodes of the cell ensures significant amplification of the PA signal, making resonant systems particularly suited for trace gas detection applications. In the design of the photoacoustic cell, particular attention must be given to optimizing the resonator geometry to achieve maximum signal-to-noise ratio (SNR). Geometrical parameters and coupling with the acoustic detector directly affect the resonator quality factor (Q), thereby influencing sensitivity and selectivity [10,11]. Additionally, the choice of materials and surface finish plays a crucial role in minimizing unwanted acoustic damping and thermal losses.

Numerical simulations and analytical models are often employed to predict resonance frequencies and mode distributions, guiding the design process before experimental validation.

In the design of the photoacoustic cell for gas detection systems, it is essential to accurately determine the key parameters that govern the acoustic behavior of the resonator—specifically, the resonance frequency ($f_{r}$) and Q. They directly influence the system sensitivity. In fact, $f_{r}$ defines the optimal modulation frequency of the light source for maximum acoustic signal generation, while the *Q* reflects the sharpness and amplitude of the resonance peak, i.e., the amplification of the PAS signal, and hence the SNR, crucial for detecting trace gas concentrations [12]. Therefore, establishing reliable geometry-dependent relationships for $f_{r}$ and Q is essential for resonator design and optimization [13].

For resonator geometries lacking analytical relationships between geometry and key parameters, the design process becomes significantly more challenging and often relies on empirical or numerical approaches. Experimental testing of multiple shapes to identify optimal configurations is both time-consuming and costly. Consequently, numerical modeling—particularly based on Finite Element Method (FEM) tools—has become the preferred approach for simulating acoustic behavior and guiding design decisions, owing to its high accuracy in handling complex geometries and boundary conditions. However, FEM-based workflows are computationally demanding, require significant technical expertise, and often rely on proprietary software with restrictive licensing. In this context, the availability of analytical or semi-empirical models, even if approximate, that explicitly relate design parameters to performance metrics remains highly valuable. Analytical and semi-empirical models can provide valuable alternatives, especially in early-stage prototyping or resource-constrained contexts, enabling faster design-space exploration and provides physical insight into the dominant factors driving device optimization [14,15]. However, closed-form solutions for $f_{r}$ and Q are typically available only for simple geometries (e.g., cylindrical or Helmholtz resonators). Although cylindrical resonators represent the most extensively studied class of photoacoustic cavities, with well-established analytical solutions for both $f_{r}$ and Q [8], their use in compact and mechanically integrated PAS systems should present intrinsic limitations. Although cylindrical resonators are analytically well established, their practical implementation introduces additional losses and boundary-condition sensitivities that limit predictive accuracy. As a result, analytical solutions derived for ideal cylinders often fail to accurately predict the behavior of practical devices without extensive empirical correction.

Additionally, more complex or compact designs, such as multi-chamber or MEMS-based resonators, require not only approximations but also advanced modeling approaches.

Several studies have addressed resonator optimization through numerical or reduced-order approaches. Niedermayer et al. [16] proposed a reduced-order modeling strategy to identify internal resonances in systems with intractable equations. Kost et al. [17] employed FEM combined with Monte Carlo algorithms to optimize open resonators for pressure enhancement at the detector [18]. Firebaugh et al. [19] numerically investigated the influence of radial dimensions on SNR, while studies in [20] analyzed the geometry of H-type resonators for gas detection, confirming the strong impact of geometrical parameters on sensitivity and Q. In the field of MEMS resonators, Steeneken et al. [21] presented a parameter-extraction methodology based on eigenfrequency simulations and matched boundary conditions.

In this context, there is an even great need for efficient and adaptable modeling tools.

Among non-conventional resonator geometries, ring-shaped and toroidal photoacoustic cells have recently attracted increasing attention due to their compactness, mechanical robustness, and favorable SNRs in practical gas-sensing implementations [4,9]. In particular, the closed-loop geometry allows for efficient acoustic energy confinement while enabling a folded propagation path within a reduced footprint, representing an advantage for portable and embedded PAS devices [9,18]. Moreover, toroidal resonators naturally suppress end effects and acoustic leakage associated with open or terminated cylindrical cavities, improving stability under operating conditions [8,22,23]. Building on this foundation, we aim to develop an analytical approach that links geometry to acoustic behavior, reducing reliance on extensive FEM simulations and accelerating resonator design.

Analytical treatments of toroidal resonators with arbitrary cross-sections have been extensively developed since the 1980s, mainly in the context of electromagnetic and microwave cavities [24]. Although rigorous, these formulations are often mathematically involved and not well suited for rapid engineering design optimization and practical parameter exploration in engineering applications. In contrast, the present work adopts a reduced-order modeling approach aimed at capturing the dominant geometry-dependent trends but with minimal computational overhead.

Our study combines FEM simulations with numerical curve fitting and compares results with established analytical solutions for cylindrical and toroidal geometries. The proposed analytical model was refined to better reproduce the expected behavior, leading to a new formulation that outperforms existing approaches. Finally, the model was validated through independent experimental measurements. The goal is to derive a simplified analytical model suitable for fast prototyping, enabling first-pass estimation of $f_{r}$ and Q during the early design phase and guiding the optimization of high-performance PAS devices.

## 2. Theoretical Background: Analytical Limits and Numerical Strategy

In gas photoacoustic sensing technology, sound waves are generated by the periodic heating of a gas resulting from the absorption of intensity-modulated light at a specific frequency $f_{m}$. When the wavelength λ of the light beam matches one of the absorption peaks of the target gas, selective absorption occurs. This enables gas concentration detection through the photoacoustic effect, since the heating of the gas molecules caused by absorption generates an acoustic wave whose amplitude is directly proportional to the concentration of the trace gas [6]. The acoustic pressure can be described within the acoustic wave theory. Under the assumption that both heat and stress remain confined to the illuminated volume, the acoustic wave can be described by the inhomogeneous Helmholtz equation, where the source term $H(\vec{r},\;\omega )$ represents the absorbed light power density in frequency domain, [10] as

(1) $$\nabla^{2}p\left(\vec{r},\;\omega \right)+k^{2}p\left(\vec{r},\;\omega \right)=-j\omega \frac{\left(\gamma -1\right)}{c^{2}}H\left(\vec{r},\;\omega \right).$$

Here, p denotes the acoustic pressure (i.e., the photoacoustic signal), k is the acoustic wavenumber, ω the angular frequency (ω=2πf), c the speed of sound, and γ the ratio of specific heat at constant pressure.

To solve this equation, the eigenmode expansion method is typically employed, which assumes that the solution can be represented as a superposition of the *n*-th orthogonal eigenfunctions associated with the closed, ideal resonator. In the Fourier domain, the solution takes the form [8,22]

(2) $$p\left(\vec{r},\omega \right)=A_{0}\left(\omega \right)+\sum_{n=1}^{N}A_{n}\left(\omega \right)p_{n}\left(\vec{r}\right).$$

Here, $p_{n}\left(\vec{r}\right)$ are the eigenmodes of the homogeneous Helmholtz equation: $\nabla^{2}p\left(\vec{r},\omega \right)+k^{2}p\left(\vec{r},\omega \right)=0,$ and $A_{n}$ the related amplitude coefficients obtained by projecting the source on the eigenmodes. When loss mechanisms, such as thermal and viscous damping, are taken into account, the eigenmode terms can be modified by introducing a loss factor expressed as the quality factor $Q_{n}$ for each eigenmode. Accordingly, the amplitude of the *n*-th eigenmode near resonance can be approximated as [8,25]

(3) $$A_{n}\propto \frac{\int_{V_{r}}H\left(r,\omega \right)p_{n}\left(\vec{r}\right)dV\;}{{(\omega }_{n}^{2}-\omega^{2})+j\frac{\omega \cdot \omega_{n}\;}{Q_{n}}}$$

where $V_{r}$ is the resonator volume and $\omega_{n}$ the angular frequency of the *n*-th eigenmode.

This method is analytically tractable only for simple geometries (e.g., cylindrical or rectangular cavities). For complex or irregular structures—such as the toroidal geometry studied in this work—closed-form expressions for the mode shape and resonant frequencies do not exist, making analytical approaches unreliable [26]. Moreover, the eigenmode expansion is typically truncated (for example to the first 8 eigenmodes), introducing inherent modeling errors. Additionally, to directly compute the acoustic pressure field, further post-processing is necessary to estimate the Q, the modal amplitudes, and the actual resonant frequency of the structure. Resonance-based PA systems, which exploit resonant amplification, require precise tuning of the modulation frequency to match the system resonance ($\omega =\omega_{r}$). Therefore, knowing the exact value of the resonant frequency is crucial for properly designing the geometry to ensure optimal operating conditions. Notably, even a small mismatch between the modulation and resonant frequencies can become particularly problematic in high-Q systems where, due to the narrow bandwidth, a small mismatch may result in a significant attenuation of the acoustic response [9].

## 3. Analytical Challenges in Toroidal Resonance Modeling

Because deriving closed-form expressions for the resonance frequencies and mode shapes of toroidal resonators is challenging, this section investigates two modeling strategies: an approximated approach based on cylindrical geometry and a model tailored specifically to toroidal structures. Both models are validated and refined based on FEM simulation results, with the aim of achieving an accurate yet computationally efficient analytical representation of the actual structure. The ultimate goal is to establish a direct, even if approximate, relationship between the resonator geometry and the photoacoustic response, enabling the prediction of resonance frequencies and Q as functions of the structural dimensions and thus streamlining the design of photoacoustic sensing systems.

### 3.1. Cylindrical Model Approximation

Owing to its rotational symmetry and closed-loop geometry, a toroidal resonator can often be approximated by an equivalent cylindrical resonator when its major radius is significantly larger than its minor radius (R≫r). In this limit, the local curvature becomes negligible, and the resulting acoustic field closely resembles that of a straight cylindrical resonator [23,24,25,26,27]. This approximation greatly simplifies the analytical modeling of resonance behavior. The toroidal cavity can be treated analogously to a cylindrical one by approximating its length by the linear length of the cylinder, and consequently $f_{r}$ and Q can be estimated using the same expressions as for a cylindrical (loop-gap) resonator, while still preserving sufficient accuracy for many applications. This approach retains the benefits of analytical solvability while offering a physically meaningful approximation of the toroidal resonator [28].

For a lossless cylindrical resonator with fully reflecting (rigid) boundaries, the intrinsic acoustic resonance frequencies can be derived by solving the wave equation in cylindrical coordinates (ρ,φ,z). The time-domain form of Equation (1) can be written as [8]

(4) $$\frac{1}{\rho }\frac{\partial }{\partial \rho }\left(\rho \frac{\partial p}{\partial \rho }\right)+\frac{1}{\rho^{2}}\frac{\partial^{2}p}{\partial \varphi^{2}}+\frac{\partial^{2}p}{\partial z^{2}}-\frac{1}{c^{2}}\frac{\partial^{2}p}{\partial t^{2}}=\frac{\gamma -1}{c^{2}}\frac{\partial H\left(\rho ,t\right)}{\partial t}.$$

Imposing that the normal velocity vanishes at the cylindrical wall (ρ=r) and that both ends of the cylinder are rigid (∂p/∂z=0), the eigenmodes can be expressed as

(5) $$p_{jmq}\left(\rho ,\varphi ,z\right)=J_{m}\left(\frac{\beta_{jm}\rho }{r}\right)cos\left(m\varphi \right)cos\left(\frac{q\pi }{L}z\right)$$

where $J_{m}$ is the Bessel function of the first kind of order m, *r* and L are the cylinder radius and length, respectively, and $\beta_{jm}$ is the j-th zero of the derivative $J_{m}^{\prime }(\rho )$. Recalling that k=ω/c and $k^{2}=k_{r}^{2}+k_{z}^{2}$, with $k_{r}=\beta_{jm}/r$ and $k_{z}=q\pi /L$ being the radial and axial wavenumbers, the angular resonance frequency of mode (j,m,q) is

(6) $$\omega_{jmq}=c\sqrt{\;{\left(\frac{\beta_{jm}}{r}\right)}^{2}+{\left(\frac{q\pi }{L}\right)}^{2}},$$

and the corresponding resonance frequency is

(7) $$f_{jmq}=\frac{c}{2\pi }\sqrt{{\left(\frac{\beta_{jm}}{r}\right)}^{2}+{\left(\frac{q\pi }{L}\right)}^{2}}.$$

In practice, if the resonator has openings or terminations, the simple geometric length L does not represent the effective acoustic length, because the sound field extends slightly beyond the physical boundaries. To account for this, an end-correction ΔL  is introduced. The fundamental resonance can then be approximated as

(8) $$f_{r}\approx \frac{c}{2\;(L+\Delta L)}.$$

The end correction can be estimated as $\Delta L≃\alpha_{c}r$, where the coefficient $\alpha_{c}$ depends on the geometry and boundary conditions (typically $\alpha_{c}\approx 0.6$ for an unflanged open end [13]).

### 3.2. Toroidal Model Approximation

Although a torus (with circular cross-section) is generated by rotating a circle around an external axis—resulting in a closed-loop geometry—its structure exhibits superficial similarities to that of a cylindrical resonator. Like the cylinder, the torus can be fully characterized by two geometrical parameters: its major radius $R_{t}$ (the distance from the center of the torus to the center of the circular cross-section) and the minor radius $r_{t}$, which is the radius of the cross-section. Because of this similarity, cylindrical models are sometimes used as a coarse approximation for the acoustic behavior of toroidal resonators providing quick estimates of the resonance frequencies [29,30].

**Figure 1:**
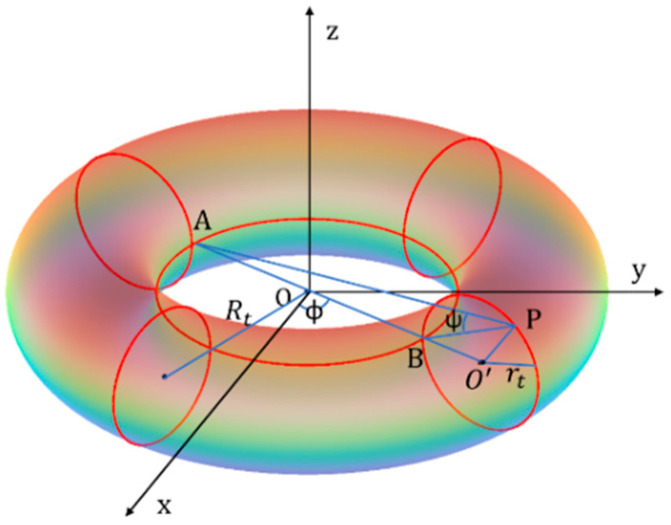
Toroidal coordinate system.

However, because of its curvature, boundary conditions, and the modal distribution, the toroidal resonator is inherently more complex. As a result, the cylindrical approximation often fails to accurately capture the modal behavior and frequency spectrum, especially for higher-order modes or when $R_{t}\;$ and $r_{t}$ become comparable. A dedicated model is therefore required to describe the acoustic properties of toroidal structures with greater precision. In this context, Janaki and colleagues in [31] proposed a theoretical model describing toroidal resonators, analyzing the mode shapes and frequency characteristics that distinguish them from their cylindrical counterparts. According to [31], the acoustic wave Equation (1) is reformulated using a variant of the toroidal coordinate system, as presented in [32,33]. The main advantage of the Janaki coordinate system, compared to the commonly adopted toroidal coordinates, is that Laplace’s equation (corresponding to the static limit of the wave equation) becomes separable, and its solutions can be expressed in terms of toroidal harmonics. Under this formulation, the wave Equation (1) becomes significantly more complex. The modified system of toroidal coordinates (σ,ψ,ϕ), with reference to Figure 1, is defined by the transformations

(9) $$x=\frac{a\;sinh\left(\sigma \right)\cos\left(\phi \right)}{cosh\left(\sigma \right)-cos\left(\psi \right)},\;y=\frac{a\;sinh\left(\sigma \right)\;sin(\phi )}{cosh\left(\sigma \right)-cos\left(\psi \right)},\;z=\frac{a\;sin\left(\psi \right)}{cosh\left(\sigma \right)-cos\left(\psi \right)},$$

where $a^{2}=R_{t}^{2}-r_{t}^{2}$. On the surface of the torus, $\sigma =\sigma_{0}\;$ satisfies the geometric condition: $\cosh\sigma_{0}=\frac{R_{t}}{r_{t}}.$ The angles ψ and ϕ correspond to the poloidal and toroidal coordinates, respectively.

The acoustic wave equation was reformulated in toroidal coordinates. After expressing the Laplacian in this system, separation of variables was applied, and the resulting differential equations were solved using power-series methods. The solutions can be expressed in terms of hypergeometric (or associated Legendre) functions. In the static limit case, these solutions reduce to toroidal harmonics, which describe the natural oscillation modes of a torus. The eigenvalues and eigenfunctions of the system have therefore been determined for arbitrary numbers of toroidal and poloidal modes and, in the large aspect-ratio limit ($R_{t}>>r_{t}$), the solutions have been shown to converge with the corresponding cylindrical ones, expressed in terms of Bessel functions.

This result is consistent with physical intuition as proposed by cylindrical geometry approximation in [34,35]. Accordingly, the corresponding eigenfrequency for the toroidal-shaped resonators is determined in [31] by the following expression:

(10) $$f_{r}(R_{t},\;r_{t})=\frac{c}{2\pi }\frac{p_{nm}}{\sqrt{{R_{t}}^{2}-{r_{t}}^{2}}}$$

where $p_{nm}$ denotes a zero of the hypergeometric function.

The models in (8) and (10), which are often used in the literature, can produce results inadequate for accurately describing real-world applications. This is primarily because they rely on ideal assumptions—such as homogeneous media—and typically neglect geometry-induced dissipative effects, which can significantly impact the system resonant behavior. In the case of toroidal resonators or other complex geometries, the confined propagation of acoustic waves and the associated geometric non-uniformities give rise to local deviations from the behavior predicted for homogeneous and unbounded media. Dissipative phenomena related to viscosity and thermal conduction at the channel walls play a significant role—especially in configurations with a small cross-sectional radius (i.e., thin torus) [36,37]. These effects are particularly pronounced near the inner surface of the toroidal channel, where strong velocity and temperature gradients are generated.

To assess the relevance of dissipative mechanisms in toroidal resonators, it is useful to consider a parameter like the surface-to-volume ratio (S/V) to capture how dissipative losses influence the modal behavior. In our case, S/V is inversely proportional to the minor radius $r_{t}$. As $r_{t}$ increases, the volume grows faster than the surface area, leading to a lower S/V ratio. Since dissipative effects, such as viscous and thermal losses, are primarily surface-related, a higher S/V (i.e., smaller $r_{t}$) enhances their impact. Therefore, optimizing $r_{t}$ is key to minimizing energy losses and improving the resonator acoustic performance [37,38].

To incorporate these effects, we consider modifying the models in (8) and (10) by replacing the speed of sound c with an effective phase velocity $c_{d}$ that more accurately reflects the wave propagation conditions within the toroidal resonator accounting for frequency dependency and that can be assumed as

(11) $$c_{d}=c\left(1-\gamma \chi \right)$$

where γ is an effective length-scale parameter accounting for thermo-viscous losses and boundary effects, while χ explicitly represents geometric confinement and is proportional to the surface-to-volume ratio of the resonator cross-section ($\chi ∼S/V\approx 1/r_{t}$). In this framework, $c_{d}$ represents a reduced-order approximation of the physical phase velocity, which in classical thermo-viscous wave propagation theory is given by $c_{ph}=\omega /Re(k)$ where the acoustic wavenumber *k* becomes complex due to dissipative effects. In confined geometries, the complex wavenumber is known to depend on the ratio between the boundary-layer thickness and characteristic transverse dimension (δ/r) which in turn affect *Re*(*k*). In the weak-dissipation regime (δ/r << 1), this dependence admits a leading-order linear approximation, thereby motivating the reduced-order form adopted in Equation (11) [39].

### 3.3. Theoretical Models and Finite-Element Simulation Comparison

To accurately compare the theoretical models introduced in the previous section with the acoustic behavior of the toroidal resonator investigated in this work, we employed Finite Element Method (FEM) simulations using COMSOL Multiphysics 5.4 software. Unlike idealized analytical approaches, FEM simulations inherently account for complex effects such as thermo-viscous losses, acoustic coupling, and geometric constraints, providing a realistic representation of the system dynamics and its physical properties.

It is worth clarifying that both the analytical formulation and the FEM simulations are developed for a toroidal resonator with circular cross-section. This choice ensures full consistency between the reduced-order analytical expressions and the numerical reference model. The FEM analysis is therefore intended as a physics-based validation of the analytical assumptions under identical geometrical conditions, rather than as a replica of the fabricated prototype geometry. The experimentally realized cells, discussed in Section 4, slightly deviate from the ideal circular cross-section due to manufacturing constraints, and this aspect is addressed in the interpretation of the experimental results.

The simulations were performed with the COMSOL Acoustics Module to derive the effective resonance frequency $f_{r}$ and Q for a toroidal structure with different size, considering operation in air at ambient temperature (25 °C) and selecting the toroidal wall of resin material. The $f_{r}$ and Q values were extracted from eigenfrequency and frequency-domain studies.

These simulations were used to assess theoretical models fidelity and to guide the development of a modified version that more accurately fits the case study. Comparing simulations with analytical predictions via model fitting, we derived a refined theoretical model which, once tuned, yielded the closest agreement and proved best suited to our design needs.

The simulations were conducted covering a range of toroidal geometries, with the major radius $R_{t}$ varying from 8 mm to 13 mm (in 1 mm increments) and the minor radius $r_{t}$ from 2 mm to 5 mm (in 0.5 mm increments). This range was chosen to comprehensively assess how geometry affects the resonator acoustic response, while remaining aligned with the target application; namely, the development of a compact, portable resonant photoacoustic gas sensor and the requirement that the dominant acoustic resonance lies within the audible frequency range (below 20 kHz), thereby ensuring efficient acoustic signal amplification [9,40].

As for the cylindrical model approximation, we approximate the axial length of the cylinder with the torus circumference, $L=2\pi R_{t}$. Including the end-correction $\Delta L=\alpha_{c}r_{t}$, Equation (8) can be rewritten as

(12) $${\hat{f}}_{r}(R_{t},\;r_{t})=\frac{c}{2(2\pi R_{t}+\alpha_{c}r_{t})}$$

where $\alpha_{c}$, which depends on the resonator geometry and the boundary conditions, is the fitting parameter adjusted to minimize the residual error between the theoretical model and the FEM simulation data. Moreover, when phase velocity correction c_d_ is included, we introduce one additional fitting parameter γ to capture residual curvature/dispersion effects. Accordingly, Equation (12) is modified to

(13) $${\hat{f}}_{r}(R_{t},\;r_{t})=\frac{c\left(1-\gamma \chi \right)}{2(2\pi R_{t}+\alpha_{c}r_{t})}$$

with γ and $\alpha_{c}$ determined by fitting to the FEM dataset.

For the toroidal model approximation, we validate the theory against the FEM results starting from Equation (10). Following the methodology in [31], the denominator of Equation (10) has been modified to account for wave propagation predominantly along the toroidal direction (along the loop, under thin torus assumption $R_{t}\gg r_{t}$).

Moreover, introducing the visco-thermal correction to the sound speed, $c_{d}$, we obtain

(14) $${\hat{f}}_{r}(R_{t},\;r_{t})=\frac{c\left(1-\gamma \chi \right)\lambda }{2\pi \sqrt{{R_{t}}^{2}+{r_{t}}^{2}}}$$

where λ, which here replaces $p_{nm}$, absorbs the mode-dependent geometric factors and boundary conditions effects.

To evaluate the agreement between the theoretical resonance frequencies and those obtained from FEM simulations, a fitting routine was implemented in the MATLAB 2023b environment. The procedure aimed to optimize the unknown parameters ($\alpha_{c}$, $p_{nm}$, γ, λ).

Both the first and the third eigenmode were considered in the evaluation. A two-dimensional nonlinear least-squares fitting procedure based on the Levenberg–Marquardt algorithm was employed to optimize the unknown model parameters by minimizing the sum of squared residuals. The residuals were defined as the pointwise relative error between the resonance frequencies predicted by the theoretical model and those obtained from FEM simulations. This formulation enables the fitting process to account for geometry-dependent discrepancies, thereby enhancing the accuracy of the models across varying resonator sizes.

To compare the performance of the models, the Average Relative Error (ARE) was computed for each fitting, defined as the average of the pointwise (n points) relative differences between the theoretical and FEM-predicted resonance frequencies:

(15) $$ARE=\frac{1}{n}\sum_{i=0}^{n}\frac{\left|f_{s-}{\hat{f}}_{r}\right|}{f_{s}}$$

Here, $f_{s}$ is the resonance frequency obtained by the FEM simulations and ${\hat{f}}_{r}$ represents instead the predicted resonance frequency. The fitting was performed across the entire set of geometric cases by evaluating all combinations of $R_{t}$ and $r_{t}$ within their respective ranges considered in the simulation.

**Figure 2:**
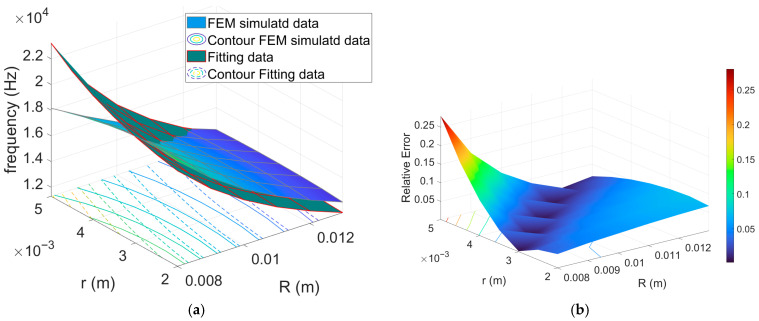
(**a**) Result of fitting obtained with model (10) for the third harmonic; (**b**) corresponding pointwise absolute relative error.

**Figure 3:**
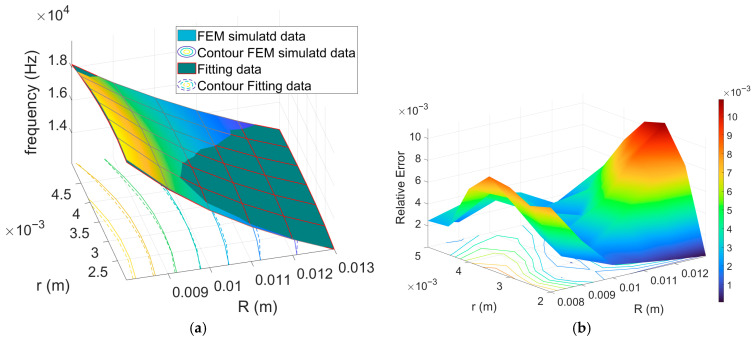
(**a**) Result of fitting obtained with model (14) for the third harmonic; (**b**) corresponding pointwise absolute relative error.

Figure 2 and Figure 3 graphically show examples of fitting results for Models (10) and (14), respectively, and their pointwise ARE concerning the third eigenfrequencies, whereas Table 1 reports the mean relative error evaluated for each model related to the first and third eigenfrequencies. As can be observed, the proposed modified forms of Equations (10) and (12) (thus models (13) and (14)) significantly improve the performance of available theoretical models. These adjustments enable the models to predict the resonance frequency with satisfactory accuracy, closely aligning with the trends observed in FEM simulations. In contrast, the unmodified models are unable to reproduce the trends predicted in the FEM simulations (see Figure 2a,b).

The comparison clearly indicates that the corrected toroidal model (Equation (14)) provides the best agreement with FEM simulations, yielding an average relative error below 1% for both the fundamental and third eigenmodes. In contrast, the simplified cylindrical approximations and the unmodified toroidal formulation exhibit larger deviations, with errors exceeding 5–10% in certain cases.

This outcome highlights that, while cylindrical-based models may capture the general resonance trends, they fail to accurately describe the more complex modal behavior induced by toroidal curvature and confinement and directly link with geometrical size. Accordingly, Equation (14) can be considered a reliable reduced-order model, bridging the gap between simple closed-form approximations and computationally expensive FEM simulations. It is worth emphasizing that the FEM simulations are not calibrated against experimental measurements, but are performed using independently defined material properties and thermo-viscous boundary conditions. In this framework, FEM results serve as a physics-based reference model. The parameter estimation procedure described above should therefore be interpreted as a model-order reduction step, aimed at embedding complex physical mechanisms into a compact analytical formulation with explicit geometrical dependence, rather than as an empirical calibration.

Beyond its impact on resonance frequency, the torus geometry additionally influences the resonator quality factor.

**Figure 4:**
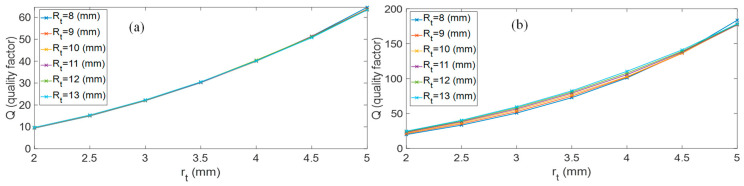
Quality factor Q estimated from FEM simulation for each major radius $R_{t}$ as a function of $r_{t}$; (**a**) first eigenfrequency, (**b**) third eigenfrequency.

The simulation results in Figure 2 and Figure 3 indicate that variations of the $R_{t}$ predominantly affect the value of $f_{r}$, which is only marginal dependent on $r_{t}$, that instead sets the cross-sectional area, governing the balance between stored acoustic energy and thermo-viscous dissipation. Because viscous and thermal losses concentrate within boundary layers adjacent to the walls, the effective dissipation scales with the surface-to-volume ratio of the cross-section, which increases as $r_{t}$ decreases. Consequently, smaller $r_{t}$ produce stronger relative damping and lower Q, while larger $r_{t}$ alleviate boundary-layer losses and yield higher Q, as highlighted by FEM simulation results reported in Figure 4. For the geometries considered, the curves obtained for different values of $R_{t}$ nearly collapse onto a single trend when plotted as a function of $r_{t}$, confirming that $r_{t}$ acts as the dominant design parameter for tailoring dissipation without substantially perturbing $f_{r}$ for the considered application case.

Guided by the trends observed in the FEM simulations, we formalize $Q=f\left(r_{t}\right)$ using a compact, semi-empirical law that reflects the leading-order thermo-viscous scaling with cross-sectional size through the parameter χ defined in Section 3.2 $(\chi =1/r_{t})$, as follows:

(16) $$Q(\chi )=\frac{\alpha }{\chi^{2}}+\beta .$$

Although a first-order scaling of the quality factor with the characteristic transverse dimension can be derived from simplified *S/V* arguments under geometric similarity, this assumption is not strictly satisfied in the present study, as we can infer from results in Figure 4. In fact, in the FEM campaign, in order to faithfully reproduce the experimental excitation conditions, the toroidal resonator is excited using a fixed localized heat source and identical boundary conditions for all considered values of the monitor; in this condition, the modal velocity and temperature gradients within the thermo-viscous boundary layers do not scale linearly with the cross-sectional size. Thermo-viscous dissipation is governed by shear and thermal diffusion within these boundary layers and becomes increasingly sensitive to the ratio between boundary-layer thickness and channel dimension, especially when the cross-section approaches the narrow-duct regime, where boundary layers partially interact and modify the effective damping behavior. This mechanism, well documented in thermo-viscous acoustics and acoustic wave propagation studies involving narrow channels [39,41], suggests the occurrence of possible deviations from simple linear scaling, due to boundary-layer overlap and non-uniform velocity profiles. Over the investigated geometric range, these effects manifest as an apparent quadratic dependence of Q  on r, which motivates the empirical formulation adopted in Equation (16).

In this formulation, α>0 and β are constants that account for the geometry- and mode-dependent gain associated with the increase of stored acoustic energy with cross-sectional areas, as well as the influence of the test conditions. Following the procedure used for the $f_{r}$ model, we estimated α and β via nonlinear least squares fitting using MATLAB lsqnonlin (Levenberg–Marquardt), by minimizing the squared relative residuals between Model (16) and the FEM results over the values of $r_{t}$.

The Model (16) was fitted separately to the datasets for the first and third eigenfrequency. The resulting estimates are $\left(\alpha ,\beta )=(2.58\;{mm}^{-2},\;-0.31\right)$ and $\left(\alpha ,\beta )=(7.16\;{mm}^{-2},\;-4.09\right)$ for the first and third eigenfrequency, respectively. The larger α at the third harmonic indicates a steeper increase of Q with $r_{t}$ at higher mode order. The negative β values are best interpreted as effective offsets that absorb radius-independent losses or modest model–measurement mismatch; they are not intended to carry standalone physical meaning outside the $r_{t}$ range explored.

**Figure 5:**
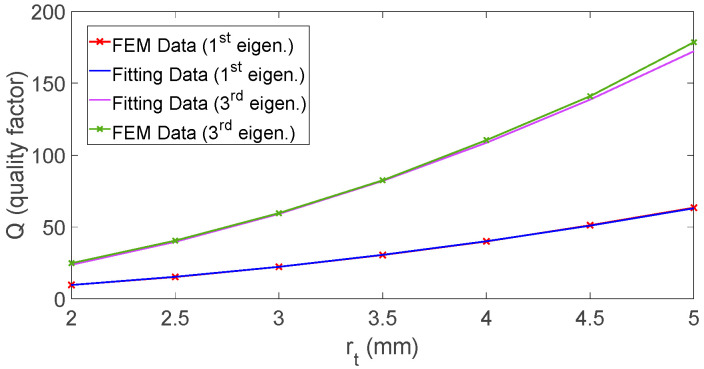
Results of fitting obtained with model (16) for the 1st and 3rd eigenmode.

Figure 5 shows the fitting results obtained by the proposed $Q(r_{t})$ model to finite-element (FEM) results for both eigenmodes. The agreement is higher in the 2–5 mm range, where the model curves capture the monotonic, convex increase of Q with increasing $r_{t}$. The relative fitting error is about 0.45% for the first eigenmode and about 0.89% for the third.

## 4. Experimental Validation

### Experimental Setup

To validate the proposed analytical and numerical models, a set of experimental measurements was performed using toroidal photoacoustic cells fabricated through a high-resolution resin 3D-printing process. The cells were designed to reproduce the optimized geometrical parameters investigated in the simulations while ensuring mechanical robustness and optical accessibility. Due to printing and assembly constraints, the toroidal channel exhibits a quasi-rectangular cross-section rather than an ideal circular one. While the overall geometry preserves the toroidal topology and the target minor radius, the internal profile slightly deviates from the circular cross-section assumed in the analytical and FEM models.

**Figure 6:**
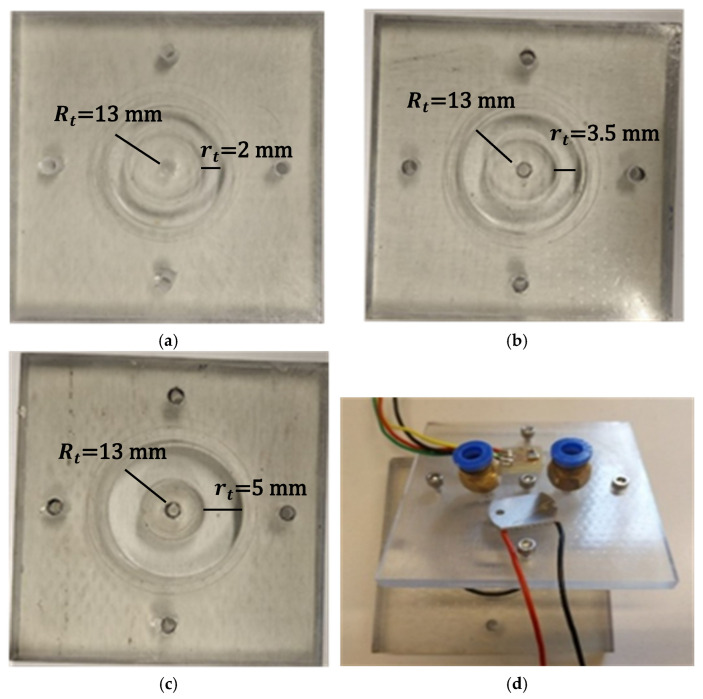
Resin-based toroidal PA cells fabricated for experimental validation; (**a**) $r_{t}$ = 2 mm, (**b**) $r_{t}$ = 3.5 mm, (**c**) $r_{t}$ = 5 mm; $R_{t}$ = 13 mm for each of the samples; (**d**) resin cell plug.

Three resin-based toroidal chambers were fabricated, all having the same major radius $R_{t}=13\;\mathrm{mm}$ and different minor radii ($r_{t}=2,\;3.5$, and 5 mm), as shown in Figure 6. The major radius was intentionally kept constant, as the numerical study indicated that the minor radius predominantly affects the quality factor, which is the most relevant parameter for resonant photoacoustic systems employing fixed-frequency signal-conditioning electronics. Under these constraints, controlling Q is more critical than further tuning the resonance frequency.

The toroidal cavity was 3D-printed as a continuous resin body and sealed on the top by a flat cover plate. This plate acted as a plug and incorporated four circular apertures: two for gas inlet and outlet, and two additional ports designed to accommodate the light source; in this case, we used an ultra violet LED (WL-SUMW SMD, Würth Elektronik eiSos GmbH & Co. KG, Deutschland) and a microphone (TDK InvenSense ICS-40800, InvenSense, Inc., San Jose, CA, USA). The same cover plate was used in all the tests, thereby using the same LED and microphone, and obtaining the same conditions in terms of LED and microphone coupling and gas delivery. The assembly provided both gas exchange and optical/acoustic access to the cavity while maintaining airtight sealing of the resonator (Figure 6d).

Measurements were carried out under controlled laboratory conditions, at ambient temperature (about 25 °C) with a continuous flow of ultrapure synthetic air at 200 mL/min through the chamber.

**Figure 7:**
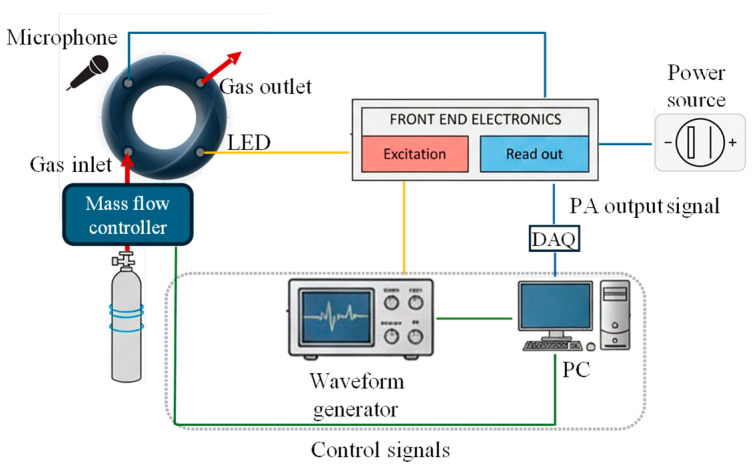
Block diagram of the PA measurement setup.

The experimental setup, depicted in Figure 7, consisted of a modulated optical source, the UV LED, coupled with the toroidal resonator such to illuminate the inner part of the torus.

The modulation frequency was provided by a function generator configured to deliver a sinusoidal waveform whose frequency was swept around the predicted eigenmodes of the resonator. In our case, we considered a 2 kHz interval centered on the first (about 4 kHz) and third (about 12 kHz) resonance frequencies predicted by the FEM simulation when $R_{t}=13\;mm$ is assumed [9]. The output signal of the generator was converted to a custom voltage-to-current conversion stage, which ensured stable current driving of the selected LED. This approach enabled precise control of the optical modulation signal amplitude and frequency, while maintaining linearity in the LED response. The modulated optical light, through the photoacoustic effect, produces a PA wave, thus, the resulting pressure oscillations are detected using a low-noise MEMS microphone (MP23AB02B, STMicroelectronics). This signal is properly amplified and filtered using the dedicated analog front-end electronics described by authors in [39] based on a mixer. The PA signal is then acquired by a 16-bit data acquisition board (DAQ: National Instruments NIPCI6224 with a maximum sampling rate of 250 kS/s) and then analyzed in frequency domain via Fast Fourier Transform (FFT) and processed and visualized on a PC running a dedicated LabVIEW 2016 virtual instrument (VI). We reconstruct the PA cell acoustic frequency response by recording the FFT magnitude of the output while letting the modulation frequency vary within a fixed 2 kHz window centered on the FEM-predicted resonance; the frequency step between consecutive excitations is adaptively selected by an optimized peak-tracking algorithm [4].

Starting from the measured frequency response, we identify the effective resonance frequency $f_{r}$ and quality factor Q by fitting an equivalent electromechanical model of the resonator using MATLAB lsqnonlin nonlinear least-squares solver (Levenberg–Marquardt). This procedure provides the experimental estimates $f_{r}^{meas}$ and $Q^{meas}$, which are used as reference values for model validation.

Modeling the device as a second-order linear lumped parameter network around the resonance frequency time-invariant (LTI) system leads to the following magnitude response (see Appendix A):

(17) $$\left|H\left(j\omega \right)\right|=\frac{2\pi fC}{\sqrt{{\left(1-{\left(\frac{f}{f_{r}}\right)}^{2}\right)}^{2}+{\left(2\xi \frac{f}{f_{r}}\right)}^{2}}},$$

where ξ is the damping ratio related to the quality factor by ξ=1/2Q. The parameters $f_{r}$ and Q are then obtained by fitting $\left|H\left(j\omega \right)\right|$ to the measured frequency response of the cells under test, while C is an equivalent capacitance related to the gas compressibility. The resonator model approximation is described in detail in Appendix A.

The experimental values $f_{r}^{meas}$ and $Q^{meas}$ are then compared with the corresponding theoretical predictions $f_{r}^{model}$ and $Q^{model}$ obtained from the proposed analytical models in Equations (14) and (16), using the parameters γ,λ,α,β previously estimated from FEM fitting in Section 3.3. This comparison is used to quantitatively assess the predictive accuracy of the proposed theoretical models.

Table 2 reports the experimentally extracted resonance frequencies together with the analytical model predictions for the first and third eigenmodes, for each value of $r_{t}$. The relative percentage error $E_{\%}$ is also reported for each case.

Importantly, no re-estimation of the analytical parameters was performed using experimental data. The comparison presented in this section is therefore a direct validation of the predictive capability of the reduced-order analytical model, whose parameters were previously identified solely from the FEM dataset.

**Figure 8:**
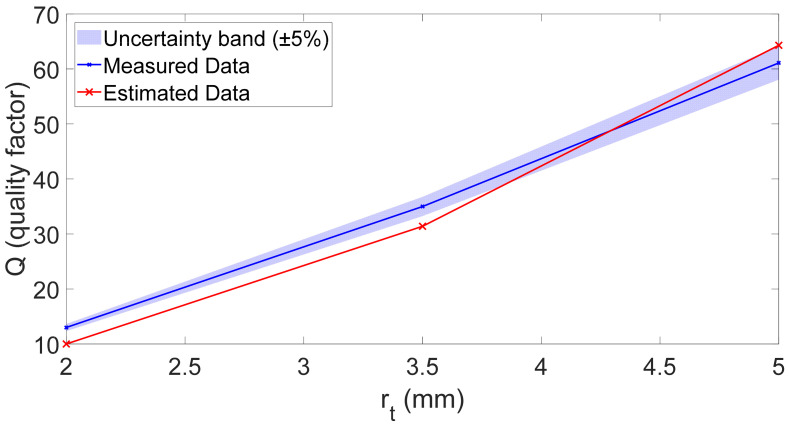
Quality factor Q versus minor radius $r_{t}$ (mm) for the 1st eigenmode. Blue line: measured data $Q_{meas}$; red line: model estimate $Q_{model}$. The shaded band represents a ±5% experimental uncertainty on the measured quality factor.

Figure 8 compares the quality factor Q predicted by the model and the experimentally measured values for the first eigenmode. As can be observed, the results obtained with the three toroidal cavities confirm the trends predicted by the FEM simulations and the analytical models, highlighting the role of the surface-to-volume ratio (S/V) in setting viscous and thermal dissipation. In particular, smaller cross-sectional radii yield larger S/V, which intensifies boundary-layer losses and reduces the quality factor; conversely, increasing the cross-sectional radius lowers S/V, mitigates dissipation, and produces sharper resonance peaks. This finding confirms the use of $r_{t}$ as a direct design lever to tune the resonator damping without substantially perturbing $f_{r}$. For the first eigenmode, the agreement is consistently good across the entire investigated range of $r_{t}$, indicating that the model captures the dominant thermo-viscous dissipation mechanisms throughout the considered size range.

**Figure 9:**
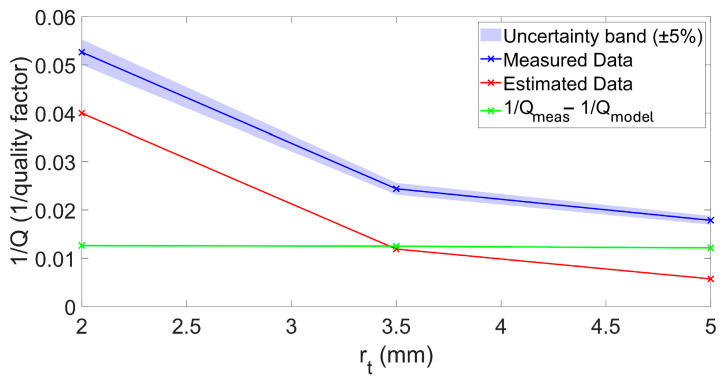
Inverse quality factor 1/Q versus minor radius $r_{t}$ (mm) for the 3rd eigenmode. Blue line: measured data $1/Q_{meas}$; red line: model estimate $1/Q_{model}$ green line: residual $1/Q_{add}$ The shaded band represents a ±5% experimental uncertainty on the measured quality factor.

In Figure 8 and Figure 9, the shaded band represents the experimental uncertainty estimated from the measurement campaign on 10 repeated measurements for each configuration. This band represents a conservative upper bound (±5%) of the overall experimental variability of the extracted quality factor Q, rather than a formal expanded uncertainty. The dominant contribution arises from fabrication-related geometric tolerances of the SLA-printed cavity. Over the investigated range, the fitted semi-empirical model indicates an approximately quadratic dependence of Q on the minor radius $r_{t}$ (i.e., $Q\propto r_{t}^{2}$). Consequently, relative deviations in $r_{t}$ up to approximately 2.5%—considering printer accuracy and process-related shrinkage of the Formlabs SLA system—may propagate into a worst-case variation approaching 5% in Q, according to first-order sensitivity analysis.

The additional variability associated with the nonlinear frequency–response fitting procedure was experimentally assessed through repeated measurements under fixed geometric conditions, yielding a maximum relative deviation of approximately 2.9%. When interpreted in terms of standard uncertainty, the geometric and fitting contributions are of comparable magnitude and lead to a combined relative standard uncertainty of approximately 4% using root-sum-of-squares aggregation. Minor contributions from acquisition electronics and measurement-chain noise were experimentally verified to be significantly smaller and are therefore encompassed within the reported conservative bound. The shaded region therefore provides a visual confidence interval, enabling a more robust and physically meaningful comparison between experimental data and model predictions.

For the first eigenmode, the agreement is generally satisfactory; however, two experimental points exhibit slightly higher Q values than predicted by the model. This behavior can be partially attributed to the fact that the fabricated channel does not have a perfectly circular cross-section. In the printed prototype, the parameter $r_{t}$ corresponds to one side of a quasi-rectangular cross-section rather than to the radius of an ideal circle. As a consequence, the effective cross-sectional area—and therefore the hydraulic radius governing thermo-viscous dissipation—is slightly larger than assumed in the analytical and FEM models.

Since the fitted law indicates an approximately quadratic dependence of Q on $r_{t}\;$ in the investigated range, small relative geometric deviations can produce amplified effects on the predicted quality factor. This effect is more pronounced for smaller cross-sections, where boundary-layer losses are more sensitive to dimensional tolerances and become progressively negligible as the channel size increases.

For the third eigenmode, as shown in Figure 9, a systematic discrepancy emerges, with the analytical model consistently overestimating the quality factor with respect to the experimental measurements (i.e., underestimating the effective dissipation). This behavior can be interpreted within the framework of additive dissipation mechanisms, for which inverse quality factors combine as

(18) $$\frac{1}{Q_{meas}}=\frac{1}{Q_{model}}+\frac{1}{Q_{add}},\;$$

where $Q_{model}$ represents the geometry-dependent thermo-viscous contribution, and $Q_{add}\;$ accounts for additional independent loss channels associated with the experimental setup (e.g., microphone acoustic loading, mounting interfaces, and electronic front-end).

It is important to emphasize that Figure 9 reports the inverse quality factor (1/Q), rather than Q itself, precisely to visualize the additive nature of independent dissipation mechanisms. The residual term $1/Q_{add}=1/Q_{meas}-1/Q_{model}$, plotted in green, exhibits an approximately constant trend over the investigated range $r_{t}=2\text{–}5$ mm. This indicates that the additional loss channel does not scale with the minor radius and is therefore not attributable to thermo-viscous mechanisms captured by the analytical model. Instead, it can reasonably be associated with setup-induced contributions, such as microphone acoustic loading, port coupling, or other geometry-independent elements of the experimental implementation.

After accounting for this constant inverse-Q contribution, the geometry-dependent trend predicted by the model aligns well with the experimental data.

It should also be noted that the additive contribution becomes visible for the third eigenmode not only because of its higher intrinsic Q, which results in a sharper and narrower resonance peak (i.e., reduced bandwidth), but also because the measurement chain employed in this frequency range differs from that used for the first mode. Although the electronic stages are not expected to introduce true acoustic losses, differences in filtering, demodulation bandwidth, and signal-conditioning parameters can affect the effective peak-shape reconstruction and therefore the extracted Q.

For the first eigenmode, the lower intrinsic Q and broader resonance peak partially mask such secondary effects.

Summarizing, the experiments and model predictions exhibit a satisfactory level of agreement, demonstrating that the proposed analytical models reliably capture the geometry-dependent behavior of $f_{r}\;$ and Q, thereby supporting their use for predictive design.

## 5. Conclusions

This work establishes a simple, reliable link between the geometry of a toroidal photoacoustic resonator and its performance metrics $f_{r}$ and Q. By combining FEM simulations with reduced-order analytical formulations and an electromechanical parameter-extraction procedure, we obtain fast, physics-informed tools that balance accuracy and computational cost. The corrected toroidal model predicts the first and third eigenfrequencies with average relative error ≲1%, consistently outperforming cylindrical approximations and uncorrected toroidal baseline models. For the quality factor, the proposed semi-empirical law captures the measured trends: increasing the cross-sectional radius lowers the surface-to-volume ratio, mitigates thermo-viscous losses, and thereby increases Q. Across three fabricated cells, the first-mode behavior is well reproduced; for the third mode, a systematic offset is consistent with an $r_{t}$-independent electrical-loss channel in the readout, which we quantify via inverse-Q superposition. These results validate the models as practical predictive-design tools, enabling rapid geometry sweeps and optimization under specified operating conditions, and provide a path to extensions that include temperature/gas-property variations, port/microphone coupling, and higher-order corrections for strongly curved or non-slender geometries. The hierarchical validation strategy adopted in this work—combining physics-based FEM modeling, reduced-order analytical formulation, and independent experimental verification—ensures both interpretability and predictive robustness.

## Figures and Tables

**Table 1:** Average relative error (ARE) of the fitting for the first and third eigenmodes across all considered models.

ARE (%)				
Eigenfrequency	Model (12)	Model (13)	Model (10)	Model (14)
1st	29.90%	14.14%	4.64%	0.77%
3rd	28.90%	13.81%	5.58%	0.43%

**Table 2:** Measured and model-predicted resonance frequencies (Hz) for the 1st and 3rd eigenmodes at each $r_{t}$. The last row reports the relative percentage error ($E_{\%}$) for each case.

	1st Eigenfrequency (Hz)			3rd Eigenfrequency (Hz)		
$r_{t}(mm)$	2	3.5	5	2	3.5	5
$f_{r}^{meas}$	4136	4166	4177	12,041	12,159	11,920
$f_{r}^{model}$	4013	4217	4207	11,903	12,055	11,923
$E_{\%}$	0.78	1.22	0.73	1.15	0.86	0.025

**Figure A1:**
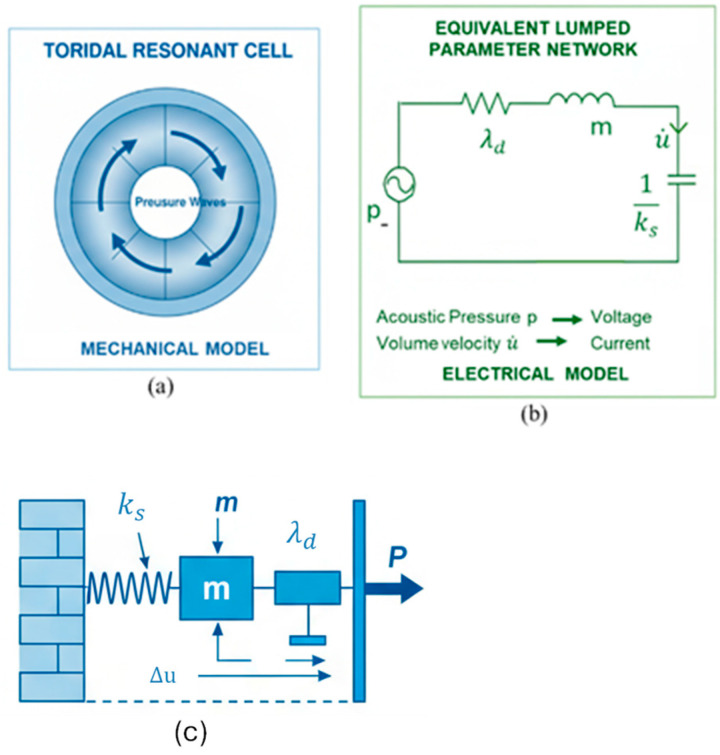
Schematic representation of the toroidal resonant cell and its equivalent electro-mechanical model. (**a**) Mechanical/acoustic view with standing pressure waves in the torus; (**b**) Equivalent lumped-parameter electrical network; (**c**) Analogue mass–spring–damper used for model fitting.

## Data Availability

The original contributions presented in this study are included in the article. Further inquiries can be directed to the corresponding author.

## Abbreviations

The following abbreviations are used in this manuscript:

FEM	Finite Element Method
PAS	Photoacoustic spectroscopy
VOCs	Volatile organic compounds
SNR	Signal-to-noise ratio
ARE	Average Relative Error
LED	Light Emitting Diode
DAQ	Data Acquisition board
LTI	Linear time-invariant

## Appendix A. Electro-Mechanical Equivalent Model of Resonator

This section presents a comprehensive lumped-parameter model of the complete resonant system. The proposed model aims to capture the essential mechanical and electrical characteristics of the device. The dynamic behavior of the system is described using a mass-spring-damper representation, as illustrated in Figure A1.

Specifically, in the equivalent electrical and mechanical circuits the acoustic mass m accounts for inertia effects (gas acceleration within the cavity), the equivalent spring $k_{s}$ represents acoustic stiffness (gas compressibility), and the damping coefficient $\lambda_{d}$ models energy dissipation due to viscous and frictional losses along the resonator walls.

Based on this representation, with acoustic pressure p as input (forcing function) and volume velocity $\dot{u}$ (structural or gas displacement rate) [42] as output, the transfer function in the Laplace domain is

(A1) $$H\left(s\right)=\frac{\dot{u}\left(s\right)}{p\left(s\right)}=\frac{1}{Z\left(s\right)}=\frac{s}{s^{2}m+s\lambda_{d}+k_{s}},$$

where $Z\left(s\right)=sm+\lambda_{d}+k_{s}/s$ is the equivalent acoustic impedance.

By mapping acoustic and physical quantities to electrical ones, the system is modeled as an equivalent series RLC circuit. In this case Equation (A1) can be written as:

(A2) $$H\left(s\right)=\frac{\dot{u}\left(s\right)}{p\left(s\right)}=\frac{sC}{s^{2}LC+sRC+1}\;,$$

with L, R, and C the electrical analogs of m, $\lambda_{d}$, and $1/k_{s}$, respectively.

For a compact and physically interpretable characterization, we express the transfer function in terms of the damping factor ξ and the resonance frequency $f_{r}$, with $\omega_{r}=1/\sqrt{LC}=2\pi f_{r}$, $\xi =(R/2)\sqrt{C/L}$, and the standard relation Q=1/2ξ [43,44]. Evaluating the frequency response at s=jω=j2πf, the magnitude of (A2) becomes:

(A3) $$\left|H\left(j\omega \right)\right|=\frac{2\pi fC}{\sqrt{{\left(1-{\left(\frac{f}{f_{r}}\right)}^{2}\right)}^{2}+{\left(2\xi \frac{f}{f_{r}}\right)}^{2}}}\;.$$

This formulation highlights the explicit dependence of the frequency response on $f_{r}$ and ξ (or, equivalently, Q) thereby providing a compact and physically insightful description of the system dynamics.
